# Endovascular iliac preparation for renal transplant using Viabahn stenting

**DOI:** 10.1016/j.jvscit.2025.102117

**Published:** 2025-12-26

**Authors:** Jennifer Canonge, Jana Hammoud, Cecile Champy, Jean Senemaud, Joseph Touma, Pascal Desgranges

**Affiliations:** a Department of Vascular Surgery, Henri Mondor Hospital, Créteil, France; b Department of Urology, Henri Mondor Hospital, Créteil, France; c Université Paris-Est Creteil (UPEC), Créteil, France

**Keywords:** Kidney transplant, Renal transplant, Stent-graft, Iliac calcification, Arterial calcification

## Abstract

Severe iliac calcifications may preclude standard renal transplantation (RT) because of hostile vascular access. We report a technique of iliac endovascular preparation using Viabahn stent-grafts (W. L. Gore & Associates) as arterial landing zones for RT in five high-risk patients. Endoclamping and arteriotomy and prosthotomy enabled direct anastomosis through the stent and artery. All grafts remained patent with no vascular complications or stenosis. This minimally invasive strategy avoids major bypass surgery and facilitates RT in patients previously considered ineligible. Although early outcomes are encouraging, strict patient selection and experienced surgical teams are essential. Larger studies are needed to validate long-term efficacy and safety.

Renal transplant (RT) is considered the gold standard treatment for patient with end-stage renal disease. The kidney is usually implanted on the external or common iliac artery. However, severe atherosclerosis is a frequent comorbidity due to advanced age, increasing prevalence of diabetes mellitus and prolonged dialysis. Historically, one technique to avoid an anastomosis on a heavily calcified artery was to perform a prior aortofemoral bypass.^1^, ^2^, ^3^, ^4^, ^5^ However, these procedures are associated with a significant rate of complications that may contraindicate subsequent RT, particularly in this fragile population. Furthermore, a non negligible proportion of these patients will not proceed to transplantation. Iliac endarterectomy during the same surgery is also described,^6^^,^^7^ but difficulty to clamp the artery may persist as difficulty to perform the endarterectomy in those arteries with medial calcifications.

This case series describes a technique using endovascular preparation with an iliac endograft as the implantation site for RT. This approach offers a minimally invasive alternative to address hostile vascular access in RT candidates, while preserving arterial integrity and reducing perioperative risks. We proposed it to fragile patients with massive and extended calcifications in whom aortic clamping was expected to be difficult, or when in situ transplantation was not feasible. The exclusion criteria were the patient's refusal after comprehensive information, small diameter arteries <6 mm, arterial stenosis due to protrusive calcifications, and prior external iliac stenting.

## Case reports

Written informed consent was obtained from all patients. Comprehensive information was provided about the surgical risks, including the potential for acute limb ischemia and long-term vascular complications after stenting.

All cases were reviewed in multidisciplinary meetings involving urologists, nephrologists, and vascular surgeons.

Iliac endovascular preparation was performed electively before RT in all cases. A Viabahn stent-graft (W. L. Gore & Associates) was deployed in the external iliac artery, with care to preserve the internal iliac artery ostium. Stent-grafts were sized 1:1. After an uneventful 30-day postoperative course and satisfactory Doppler ultrasound monitoring, the patients were listed for RT.

**Fig 1:**
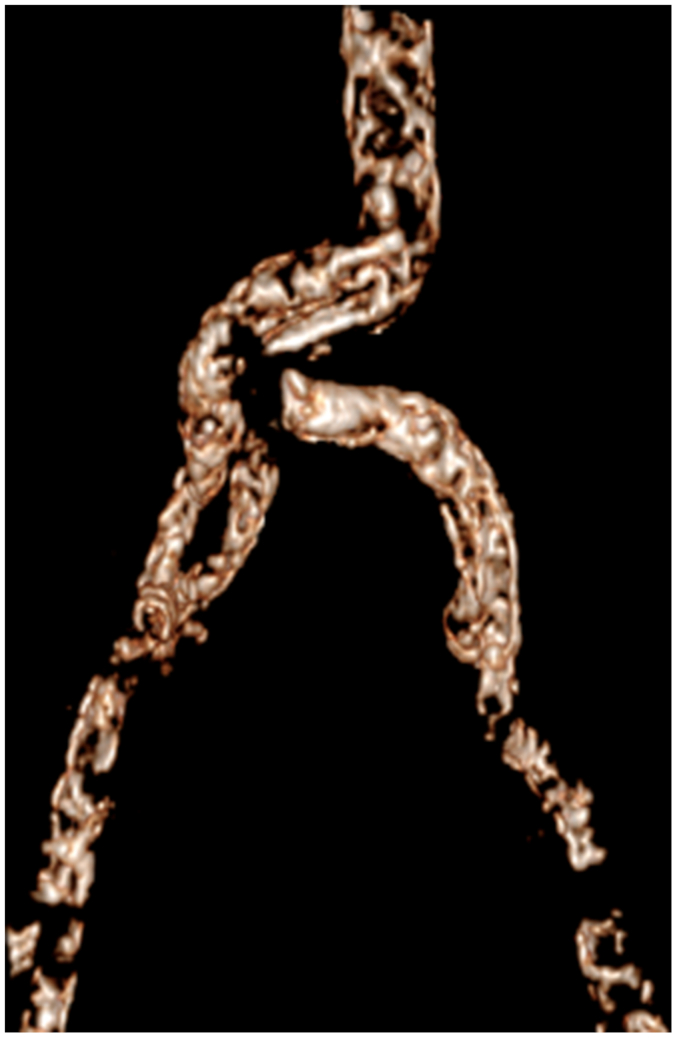
Three-dimensional scan reconstruction of severe iliac calcifications.

**Fig 2:**
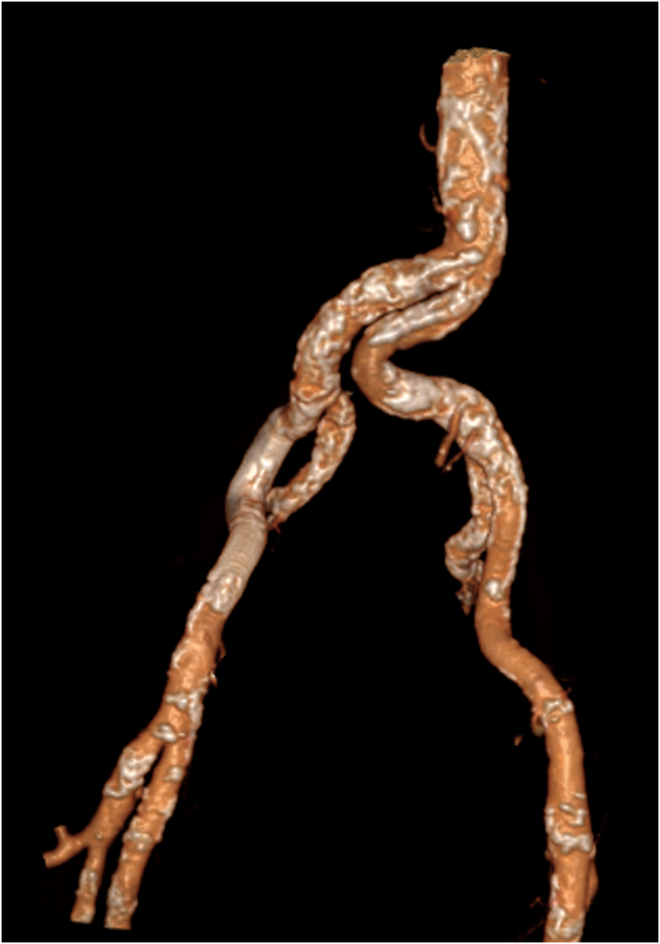
Three-dimensional scan reconstruction after Viabahn stent-graft implantation for iliac preparation.

**Fig 3:**
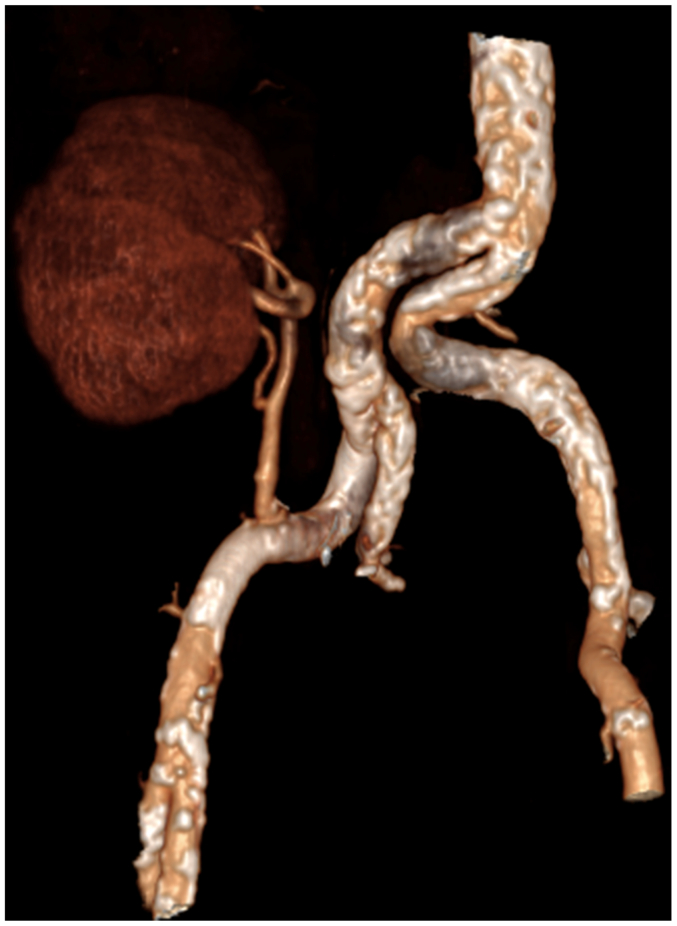
Three-dimensional scan reconstruction after renal transplant on the Viabahn stent-graft for severe iliac calcifications.

**Fig 4:**
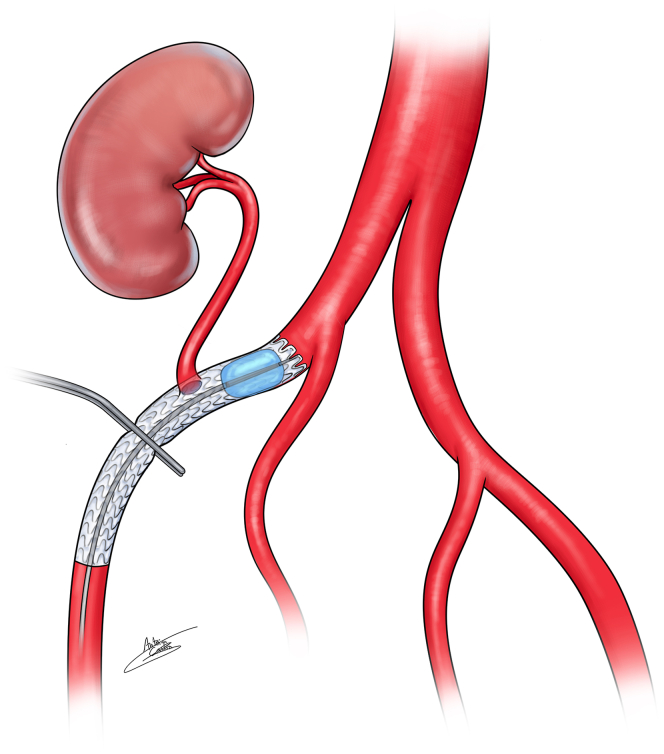
Explanatory illustration of the endoclamping and clamping technique with the Viabahn stent-graft.

The transplant procedures were performed collaboratively by a urologist and a vascular surgeon. The vascular surgeon performed the arterial anastomosis (Fig 1, Fig 2, Fig 3, Fig 4).

### Case 1

A 58-year-old male patient with a long history of dialysis was considered for RT but was deemed ineligible for standard transplant due to circumferential iliac calcifications. An 8 mm × 15 cm Viabahn stent-graft was implanted in the external iliac artery without complication.

Two and a half years later, RT was performed. After heparinization, proximal endoclamping was achieved using an 8-mm balloon via an ipsilateral retrograde access. The balloon was initially positioned under fluoroscopic control without inflation. After positioning the kidney and performing the venous anastomosis, the balloon was inflated without fluoroscopic control. The distal part of the stent was clamped using a standard metal clamp. A longitudinal arteriotomy of less than 10 mm with partial decalcification was performed. A prothotomy was also performed by cutting one stent ring with Potts scissors to allow for anastomosis. A side-to-end (lateroterminal) anastomosis was carried out using 5.0 Prolene, ensuring that each stitch passed through both the stent and the arterial wall.

After arterial and venous anastomosis, balloon angioplasty of the distal stent segment (where the clamp had been placed) was systematically performed despite no visible stenosis. An angiography with 5 mL of contrast was completed. The ureteral anastomosis was then performed.

The procedure duration was 195 minutes. Immediate, 1-month, and long-term Doppler ultrasound examinations showed satisfactory arterial and venous flow and iliac axis patency. Postoperative recovery was uneventful, and the transplant remained functional at 60 months. The last follow-up creatinine clearance was 31 mL/min.

### Case 2

A 55-year-old female patient with a diabetic nephropathy, on dialysis for 3 years and with a history of arteriovenous fistula failure, also presented circumferential iliac calcifications and was deemed ineligible for standard RT. A 7 mm × 10 cm Viabahn stent-graft was placed without complication. Six months later, the RT was performed using the same technique as in case 1 including endoclamping with a 7-mm balloon. The total procedure time was 240 minutes. Postoperative, 30-day, and 6-month follow-up Doppler ultrasound examinations were satisfactory. The postoperative course was uneventful, and the kidney graft remained functional at 7 months. Unfortunately, the patient died of nonvascular cause 7 months after transplantation. The last follow-up creatinine clearance was 85 mL/min.

### Case 3

A 63-year-old man with atherosclerotic nephropathy, not yet on dialysis, underwent endovascular iliac preparation due to circumferential calcifications. A 10 mm × 10 cm Viabahn stent-graft was successfully implanted. Three and a half years later, RT was performed. After heparinization, proximal endoclamping was performed using a 10-mm balloon. The rest of the procedure followed the same steps as in previous cases. The procedure duration was 203 minutes. Postoperative and follow-up imaging (up to 8 months) confirmed good anastomotic and iliac flow. The transplant remains functional. The last follow-up creatinine clearance was 45 mL/min.

### Case 4

A 70-year-old man with renal insufficiency due to diabetes (not yet on dialysis) and circumferential aortoiliac calcifications underwent endovascular preparation using a 7 mm × 5 cm Viabahn stent-graft. Three months later, RT was performed using the same technique as in prior cases. A balloon rupture occurred during arteriotomy, requiring replacement. The total procedure time was 300 minutes. The postoperative course was uneventful, and the kidney transplant remains functional at 4 months. Doppler ultrasound results were satisfactory. The last follow-up creatinine clearance was 67 mL/min.

### Case 5

A 70-year-old woman with a history of nephrectomy for cancer and on dialysis since 2018 underwent iliac stenting with an 8 mm × 7.5 cm Viabahn stent-graft. RT was performed 3 months later using the same approach. A reintervention was necessary on postoperative day 11 to evacuate a compressive perirenal hematoma. No other complications occurred. The procedure duration was 300 minutes. At 30 days, imaging confirmed good vascular results and transplant function. The last follow-up creatinine clearance was 32 mL/min.

## Discussion

We report our preliminary experience using endovascular iliac preparation as an alternative to bypass surgery in RT candidates with severe vascular calcifications. This technique appears feasible and reproducible, and allows RT access in patients previously deemed ineligible. Avoiding major vascular reconstruction may reduce complications in this fragile population, especially considering the number of these patients who will never receive a transplant.

Despite the apparent simplicity, we recommend strict patient selection and performance by experienced surgeons because of technical challenges, particularly when suturing through both calcified arteries and stent-grafts. Decalcification, if necessary, must be cautious to prevent arterial tear. Slagter et al^8^ described RT implantation on a stented artery using EVERPOINT polypropylene sutures, which may help penetrate calcifications. They also reported the use of a bovine patch for angioplasty before anastomosis, which we did not find necessary in our series. They encountered a balloon rupture during arteriotomy after endoclamping, which also happened to us; we managed it by using another balloon. Matillon et al^9^ described endovascular preparation using a custom-made stent-graft, illustrating the growing interest in alternatives to surgical bypass. Our technique offers advantages such as the availability of the Viabahn stent-graft and its relatively low cost compared with a custom-made device. It is also low-profile, avoiding the need for large 16F sheaths in narrowed, calcified vessels. The Viabahn stent-graft was deliberately chosen in the context of planned RT to permit an easy clamping and section of stent struts. We started to use it as iliac preparation for RT after our experience in hybrid surgery to treat iliofemoral occlusive diseases.^10^

Although this approach may enable RT access for previously excluded patients, careful evaluation of life expectancy is essential. Kidney selection is also critical; ideally, the graft should have a single artery to avoid multiple anastomoses. Although we are still on the learning curve, operative time must be carefully monitored, and appropriate precautions are required to avoid prolonged warm ischemia.

No occlusion, false aneurysm, or stenosis was observed at the renal or iliac level in this series. No patient experienced leg symptoms. However, larger studies and long-term follow-up are required to confirm the safety and efficacy of this approach.

## Conclusions

Severe calcifications are more and more frequent in RT candidates and may exclude patients from transplantation. This endovascular iliac preparation using a Viabahn stent-graft seems to offer satisfactory results avoiding the complication rates of aortofemoral or iliofemoral bypass. Larger studies are mandatory to ensure security and efficacy.

## Funding

None.

## Disclosures

None.
